# A Novel Inflammatory Dendritic Cell That Is Abundant and Contiguous to T Cells in the Kidneys of Patients With Lupus Nephritis

**DOI:** 10.3389/fimmu.2021.621039

**Published:** 2021-02-17

**Authors:** Samir V. Parikh, Ana Malvar, John Shapiro, James M. Turman, Huijuan Song, Valeria Alberton, Bruno Lococo, Juan M. Mejia-Vilet, Sethu Madhavan, Jianying Zhang, Lianbo Yu, Anjali A. Satoskar, Dan Birmingham, Wael N. Jarjour, Brad H. Rovin, Latha P. Ganesan

**Affiliations:** ^1^Division of Nephrology, The Ohio State University Wexner Medical Center, Columbus, OH, United States; ^2^Nephrology Unit, Hospital Fernandez, Buenos Aires, Argentina; ^3^Department of Pathology, Hospital Fernandez, Buenos Aires, Argentina; ^4^Department of Nephrology and Mineral Metabolism, Instituto Nacional de Ciencias Medicas y Nutricion Salvador Zubiran, Mexico City, Mexico; ^5^Department of Biomedical Informatics, The Ohio State University, Columbus, OH, United States; ^6^Division of Rheumatology and Immunology, The Ohio State University Wexner Medical Center, Columbus, OH, United States

**Keywords:** inflammatory dendritic cells, lupus nephritis, kidney, autoimmunity, adaptive immune response, T cells, SLE

## Abstract

The mechanisms that promote local inflammatory injury during lupus nephritis (LN) flare are largely unknown. Understanding the key immune cells that drive intrarenal inflammation will advance our knowledge of disease pathogenesis and inform the development of new therapeutics for LN management. In this study, we analyzed kidney biopsies from patients with proliferative LN and identified a novel inflammatory dendritic cell (infDC) population that is highly expressed in the LN kidney, but minimally present in healthy human kidneys. During an agnostic evaluation of immune transcript expression in the kidneys of patients with proliferative LN, the most abundantly overexpressed transcript from isolated glomeruli was *FCER1G*, which encodes the Fc receptor gamma chain (FcRγ). To identify the cell types expressing FcRγ that infiltrate the kidney in LN, studies were done on kidney biopsies from patients with active LN using confocal immunofluorescence (IF) microscopy. This showed that FcRγ is abundantly present in the periglomerular (PG) region of the kidney and to a lesser extent in the tubulointerstitium (TI). Further investigation of the surface markers of these cells showed that they were FcRγ^+^, MHC II^+^, CD11c^+^, CD163^+^, CD5^−^, DC-SIGN^+^, CD64^+^, CD14^+^, CD16^+^, SIRPα^+^, CD206^−^, CD68^−^, CD123^−^, CD3^−^, and CD11b^−^, suggesting the cells were infDCs. Quantification of the infDCs showed an average 10-fold higher level of infDCs in the LN kidney compared to the healthy kidneys. Importantly, IF identified CD3^+^ T cells to be adjacent to these infDCs in the PG space of the LN kidney, whereas both cell types are minimally present in the healthy kidney. Thus, we have identified a previously undescribed DC in lupus kidneys that may interact with intrarenal T cells and play a role in the pathogenesis of kidney injury during LN flare.

## Introduction

Lupus nephritis (LN) is a severe complication of systemic lupus erythematosus (SLE) that is associated with considerable morbidity and mortality. Up to 30% of patients with LN progress to end-stage kidney disease (ESKD) (1). There are no specific United States Food and Drug Administration (FDA) approved therapies to treat LN, and the current therapies produce suboptimal response rates with considerable cytotoxicity (2). We and other investigators have been exploring the molecular pathology of the kidney during active LN to better understand the pathogenesis of kidney injury in LN and pathways that may be specifically targeted to treat LN.

During the course of an agnostic evaluation of transcript expression in laser microdissected kidney tissue from clinical LN biopsies, we found that the most abundantly overexpressed transcript in the glomerular compartment was *FCER1G*, encoding the Fc receptor gamma chain (FcRγ). It was assumed that this transcript reflected immune cells infiltrating the kidney during active LN, and this work was undertaken to identify the cell types represented by the overexpressed FcRγ. Thus, in this study, transcriptomic findings were used to guide confocal immunofluorescence (IF) studies to characterize the major infiltrating immune cells present in the kidney during LN flare. We identified a unique population of FcRγ-expressing inflammatory dendritic cell (infDC) that resides in the periglomerular (PG) space and adjacent to CD3^+^ T cells signifying a potential cross-talk between infDC and T cell populations.

## Materials and Methods

### Experimental Design

The purpose of this work was to perform transcriptional analysis and IF on kidney biopsies done at the LN flare to identify the major infiltrating immune cells present in the kidney at the time of the LN flare. Transcriptomic analysis was performed on kidney biopsies obtained at the LN flare from 58 patients with proliferative (Class III/IV ± V) LN between 2007 and 2013. Archival biopsies were used after clinical testing was completed. Laser capture microdissection (LCM) was performed, and glomeruli and tubulointerstitium (TI) were isolated separately. Preimplantation living donor kidney biopsies (*n* = 10) served as healthy controls (HCs) and were analyzed in parallel with LN biopsies. The same nephrologist (A.M.) treated all the patients, and one experienced nephropathologist (V.A.) read kidney biopsies. Hospital Fernandez (Buenos Aires) ethics board and The Ohio State University institutional review board approved the investigation of the kidney biopsies.

### RNA Extraction and Analysis

The biopsies used for transcriptomic analysis were fixed in formalin and paraffin-embedded (FFPE). From the paraffin blocks, 10 μm sections were cut from each biopsy. After deparaffinization, all available glomeruli and TI were separated by laser microdissection (PALM MicroBeam, Zeiss Labs, Bernried, Germany), captured, and digested with proteinase K. DNA was removed with DNase. RNA was precipitated, extracted with RNeasy MinElute spin columns (Qiagen, Redwood City, CA, USA), and eluted in RNase-free water. Transcript expression was analyzed from 250 ng of extracted RNA using the NanoString nCounter platform and the GX human immunology transcript panel [NanoString Technologies, Seattle, WA, USA; (3–5)]. The human immunology panel v2 consisted of 579 immune response genes, 6 positive control genes, and 6 negative control genes. A complete list of these genes can be found in the earlier publication from our group (6).

For confocal IF microscopy, frozen kidney biopsy tissues from four patients with active Class IV LN were obtained from the Ohio State Nephropathology Biorepository. Three frozen nephrectomy samples were used as HC. The nephrectomies were performed in patients with renal cell carcinoma. Tissue obtained for analysis was sectioned away from the cancer tissue. The surrounding tissue used for analysis appeared healthy by histologic analysis. Nephrectomies were used as controls because frozen samples were needed, and we did not have frozen transplant donor tissue stored in our biorepository.

### Antibodies

The primary antibodies (Abs) used for IF are all listed in Supplementary Table 1. The antibodies used in this study were validated for IF by either using human lymph node or using human liver as positive control (data not shown). The isotype controls used are ChromoPure normal rabbit IgG, normal mouse IgG (Jackson ImmunoResearch, West Grove, PA, USA), mouse IgG1κ (BioLegend, San Diego, CA, USA), and mouse IgG2bκ (Jackson ImmunoResearch). The secondary antibodies used for IF were goat F(ab′)2 anti-mouse IgG 488 (Jackson ImmunoResearch) and goat anti-rabbit IgG 568, goat anti-rabbit IgG 488, and goat anti-rabbit IgG 647 from Invitrogen (Thermo Fisher Scientific, Waltham, MA, USA).

### Immunofluorescence

Frozen nephrectomy and LN kidney biopsies were sectioned (5 μm section per slide), fixed in 4% paraformaldehyde-phosphate buffered saline (PBS) for 15 min at room temperature, and washed with PBS (with 0.02% sodium azide). The sections were blocked with 5% milk in PBS, followed by incubation with the primary Ab overnight. After three washes with PBS for 1 h, the sections were incubated with fluorescently tagged secondary Abs for another hour at room temperature, and nuclei were stained with DAPI (100 ng/ml) for 10 min. The sections were then mounted with Prolong Gold (Invitrogen) under coverslips. Control Abs refer to the list of isotype Abs with their respective secondary Ab. The images were obtained using an Olympus FluoView 1000 Laser Scanning Confocal microscope equipped (Olympus Corp., Tokyo, Japan) with a spectral detection system for a finer separation of fluorochromes (FV1000 spectra) along with ×60 oil immersion lens at room temperature.

### Quantitative Microscopy

The expression level of infDC in LN and HC kidneys was quantified from images that were stained for infDC using anti-CD163. The total intensity of CD163 based on the infDC expression was calculated using ImageJ software (National Institutes of Health, Bethesda, MD, USA). CD163 intensity was obtained after subtracting the background fluorescence from the isotype plus secondary Ab-stained images and by measuring the area and the mean fluorescence intensity of the green pixels emanating from infDC using the CD163 antibody as described earlier (7).

### Statistical Analysis

For transcriptomic analysis, descriptive statistics are presented as mean ± standard deviation or as a percentage. For clinical variables, *t*-tests, anova, or Wilcoxon rank sum tests were applied as appropriate. For categorical clinical variables, Fisher's exact test was used. For nanostring data, raw counts were normalized to the positive spike-in controls and then log2 transformed. To reduce technical bias, the genes with an expression level below the mean plus two standard deviations of the negative controls were filtered out. Quantile normalization was applied to the remaining transcripts (522 for glomeruli and 502 for TI). Glomerular samples were analyzed separately from TI samples. To determine differential expression, glomerular and TI transcript expression from the LN kidneys were compared to the controls. A 1.5-fold change and *p* < 0.05 were necessary for a transcript to be considered differentially expressed.

For statistical analysis of confocal microscopy, a two-tailed Student's *t*-test was used for two-group comparison, and *p* < 0.05 was considered significant. All analyses were run using Origin Pro version 2020 (OriginLab Corp., Northampton, MA, USA).

## Results

### Transcriptomic Analysis of Kidney Biopsies at LN Flare Reveals Significant Overexpression of *FCER1G* in the Glomeruli and TI at LN Flare

**Figure 1:**
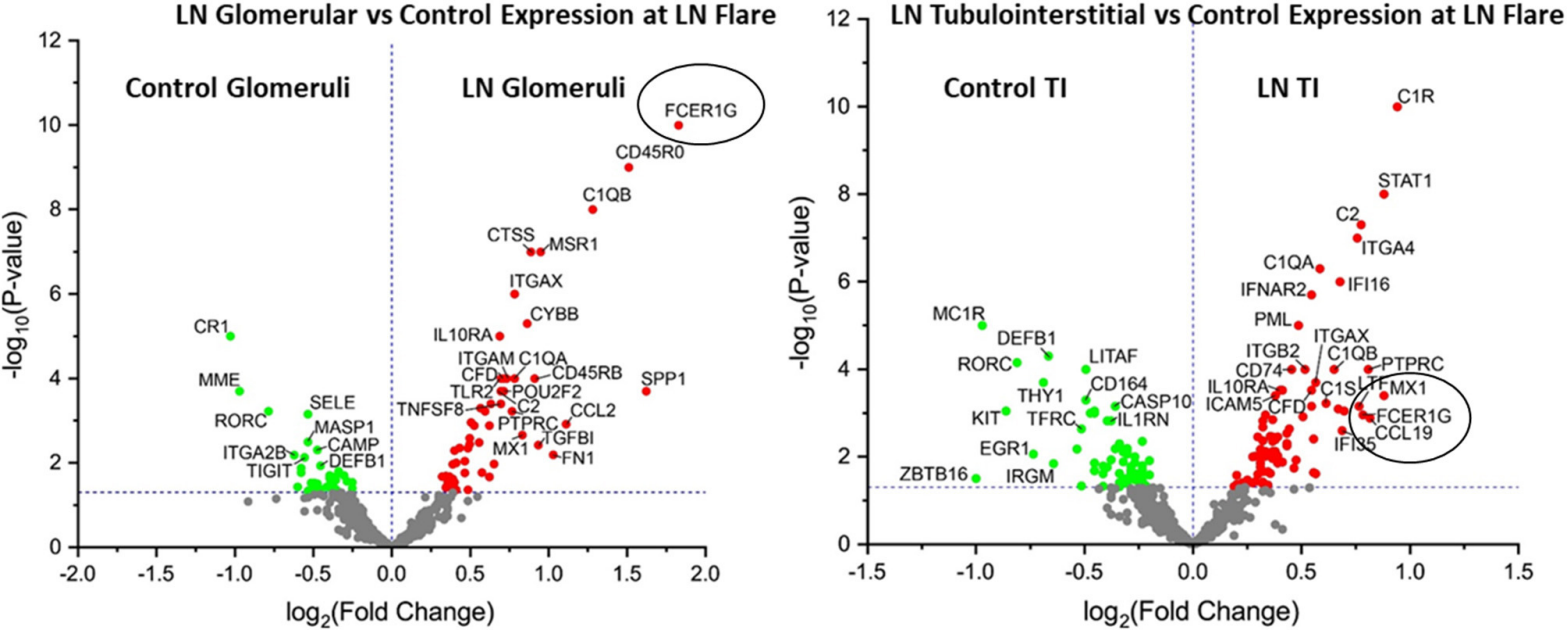
*FCER1G* is one of the top differentially expressed transcript in patients with lupus nephritis (LN). Volcano plot showing expression of *FCER1G* and other top differentially expressed transcripts in human LN glomeruli (G) (left) and tubulointerstitium (right) at the LN flare compared to healthy controls (HCs).

We performed transcriptomic analysis on RNA isolated from glomeruli and TI using LCM from the kidney biopsies obtained at proliferative LN flare (*n* = 58). Preimplantation living donor kidney transplant biopsies were used as HCs (*n* = 10). Transcriptomic analysis for 579 immune transcripts showed *FCER1G* to be the most significantly overexpressed glomerular transcript [Fold Change (FC): 3.6, *p-*value (*p*): 1E-10] but was also significantly overexpressed in the TI (FC: 1.7, *p* = 0.001) at the LN flare (Figure 1 and Supplementary Table 2) compared to HC. Additionally, the *FCER1G* expression correlated with the NIH histologic disease activity index (*r* = −0.43, *p* = 0.01).

### Kidney FcRγ Is Highly Expressed at LN Flare and Confined to the Periglomerular Region

**Figure 2:**
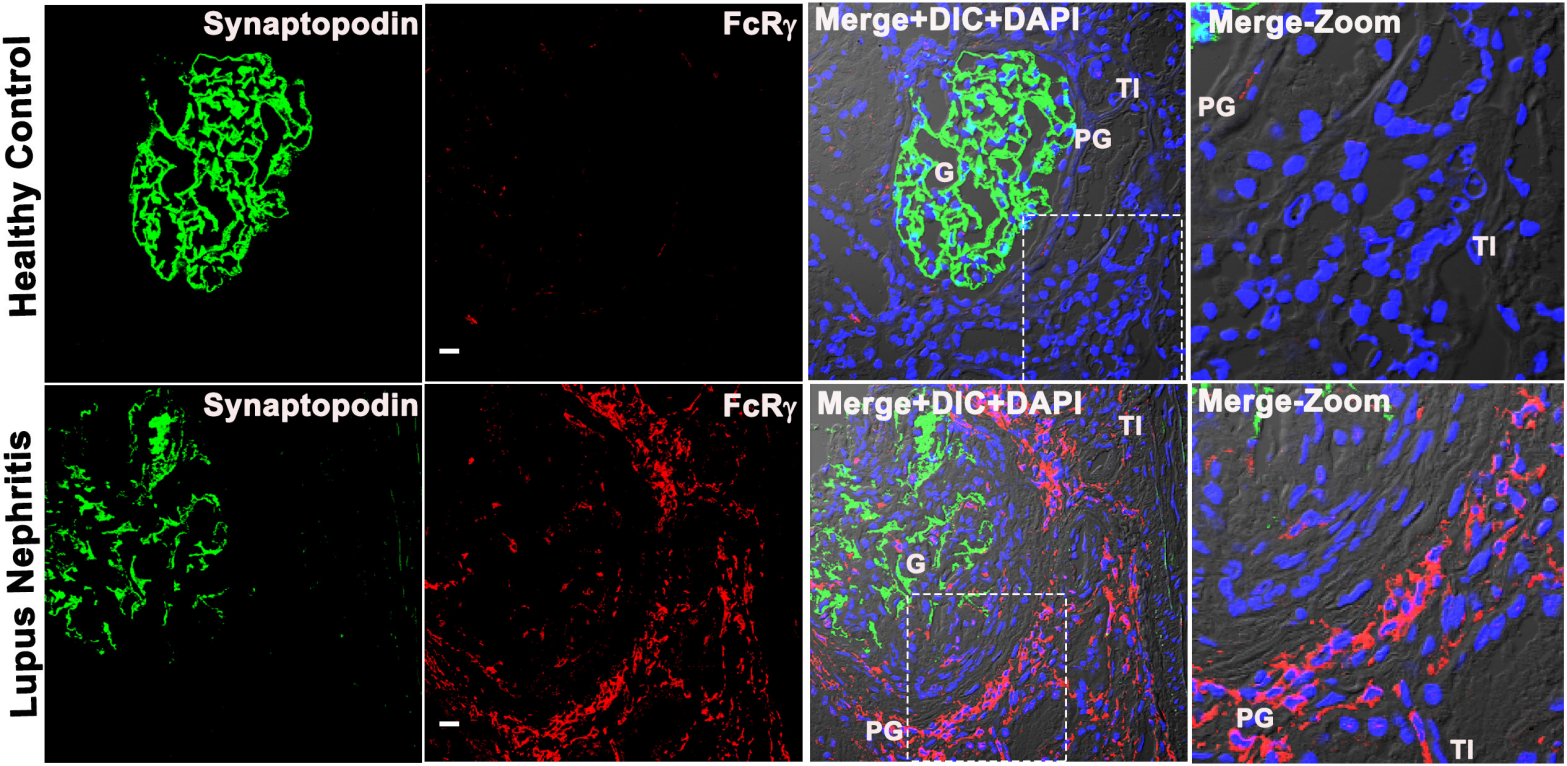
Fc receptor gamma chain is highly expressed in the patient's kidney with LN, and its expression is confined to the periglomerular (PG) region. Three color confocal IF image that is representative of 5 human LN kidney showing the expression of FcRγ (bottom row) in the PG region of the kidney and no expression in the representative of the three HC kidneys (top row). The third column shows merged images of the first 2 columns, i.e., synaptopodin (podocyte marker in green) was used as a landmark for G in the first column, FcRγ (red) in the second column plus differential interference contrast (DIC) microscopy and DAPI staining of nuclei (blue). The fourth column shows the zoomed image of the third column highlighted using a dotted square. The scale bar in the second column represents 20 μm.

To characterize the immune cell that expresses *FCER1G*, we analyzed the FCER1G encoded protein, Fc receptor gamma chain [FcRγ; (8)] in human LN, and HC kidney tissue by IF microscopic analysis using a specific antibody. The IF analysis revealed that FcRγ is minimally expressed in glomeruli (identified by the podocyte marker, synaptopodin), but abundantly expressed in the PG and TI regions of the LN flare biopsies. The cells expressing FcRγ seemed to have a typical appearance of cellular infiltrate in the PG region, microscopically (Figure 2). The IF analysis demonstrated that during LN flare, the PG region is expanded due to the presence of cellular infiltrate, whereas the PG region is thin and without cell infiltration in HC as seen in the merged images of Figure 2 and Supplementary Figure 4. Most importantly, we saw a weak-to-nil expression of FcRγ in LN and HCs using isotype antibody controls that were processed simultaneously (data not shown). These data support the transcriptomic findings that *FCER1G* codes FcRγ and is abundantly overexpressed in proliferative LN flare.

### Macrophage Markers CD11b and CD68 Did Not Colocalize With FcRγ in Human LN Kidney

**Figure 3:**
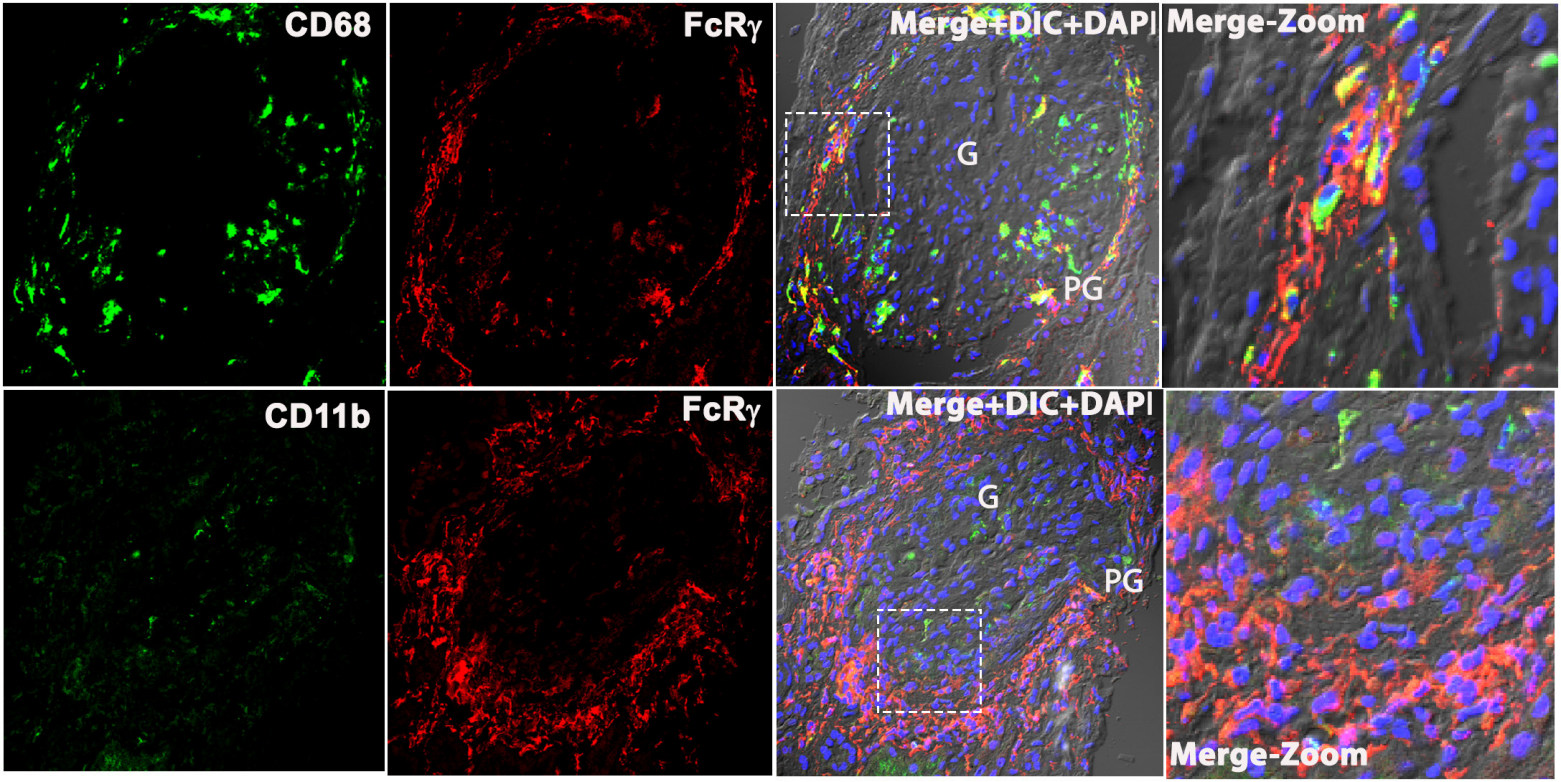
Macrophage markers CD68 and CD11b does not colocalize with FcRγ in the patient's kidney with LN. Three color confocal IF image that is representative of the 3 human LN kidneys showing no colocalization of CD68 (green) with FcRγ (red) (top row) in the PG region in the kidney and CD11b (green) with FcRγ (red) (bottom row). The third column shows merged images of the first 2 columns plus DIC and DAPI staining of nuclei (blue). The fourth column shows the zoomed image of the third column highlighted using a dotted square.

Based on prior descriptions of interstitial leukocytes in LN (9–11), we predicted the FcRγ expressing cell to be a macrophage. We dual stained biopsy sections with anti-FcRγ antibodies FcRγ antibodies against macrophage markers CD68, CD206, and CD11b. Unexpectedly, staining for M2-specific macrophage markers, CD11b and CD206 [(12); data not shown], showed weak-to-nil expression in the LN kidney. Meanwhile, the pan-macrophage marker CD68 (13) showed glomerular expression; thus, suggesting M1 macrophages were found primarily within the glomeruli. However, CD68 staining did not coincide with PG and TI staining for FcRγ (Figure 3). These data demonstrate that FcRγ expressing cells in the kidney are not macrophages. Although antibodies against CD206, CD68, and CD11b gave a weak or no signal in the LN kidney, they did stain Kupffer cells in healthy human liver robustly, confirming the reliability and specificity of the antibodies [(14); data not shown].

### FcRγ Colocalized With Conventional Dendritic Cell Marker CD11c and MHCII in LN Kidney

**Figure 4:**
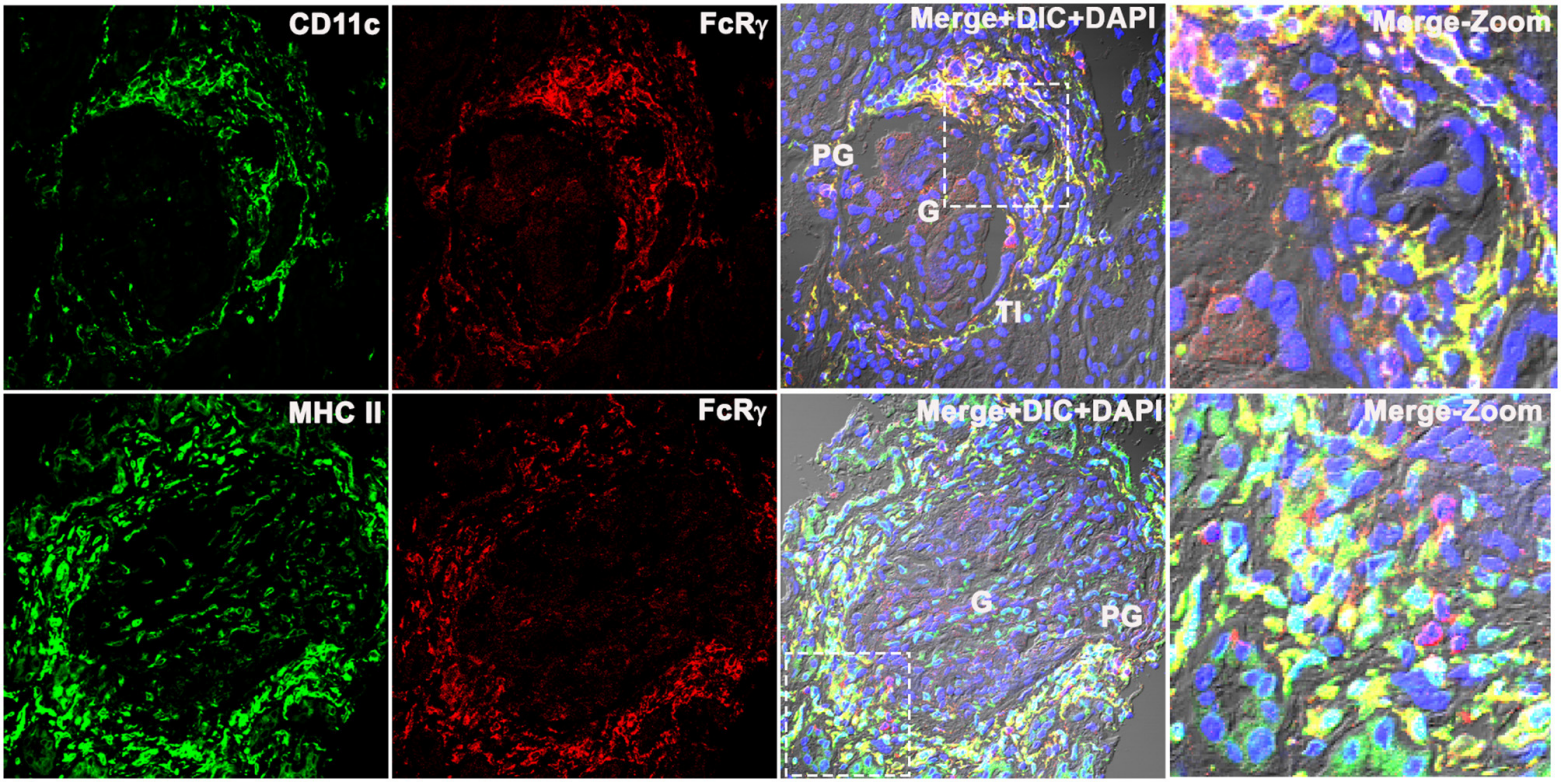
Fc receptor gamma chains colocalize with dendritic cell markers CD11c and MHCII in human LN kidney. Three color IF images that are representative of the 3 human LN kidneys showing colocalization of CD11c (labeled using anti-CD11c antibody followed by Alexa-488 dye conjugated secondary antibody that shows green emission color in confocal microscopy (green) (top row) with FcRγ labeled using anti-FcRγ antibody followed by Alexa-594 conjugated secondary antibody that shows red emission color in confocal microscopy (red). The bottom row shows the colocalization of MHC II labeled using anti-CD11c antibody followed by Alexa-488 dye conjugated secondary antibody that shows green emission color in confocal microscopy (green) with FcRγ labeled using anti- FcRγ antibody followed by Alexa-594 conjugated secondary antibody that shows red emission color in confocal microscopy (red). The third column shows the colocalization of red and green as yellow in the first 2 columns along with DIC and DAPI staining of nuclei (blue). The fourth column shows the zoomed image of the third column highlighted using a dotted square.

To determine whether the FcRγ expressing cell was a dendritic cell (DC), three-color IF microscopy was done using the conventional DC marker CD11c and MHCII that is known to be highly expressed in all human DCs (15, 16), in addition to the other myeloid cells. The qualitative analysis demonstrated that the FcRγ expression (labeled using anti-FcRγ antibody followed by Alexa-594 dye conjugated secondary antibody (Invitrogen) that showed red emission color in confocal microscopy) colocalized with both CD11c and MHCII. CD11c/MHCII were labeled using CD11c/MHC II antibody followed by Alexa-488 dye conjugated secondary antibody (Invitrogen) showed green emission color in confocal microscopy (Figure 4). Colocalization resulted in a yellow color reflecting the presence of both fluorescing FcRγ (red) and CD11c/MHCII (green) to be co-occurring in the same location/cell in the XY dimension (Figure 4). The staining pattern of CD11c matched with FcRγ (Figure 4) in PG and TI region, and neither antibody stained within the glomeruli. In line with CD11c, MHCII (Figure 4, bottom row) also stained strongly in PG, colocalizing with FcRγ. But, in addition to strong PG staining, MHCII also demonstrated weak staining within the glomerulus, suggesting the presence of a weak MHCII expressing glomerular cell that may be a myeloid cell.

### FcRγ Did Not Colocalize With the Plasmacytoid Dendritic Cell Marker CD123, but Colocalized With Monocyte-Derived Dendritic Cell Marker DC-SIGN

**Figure 5:**
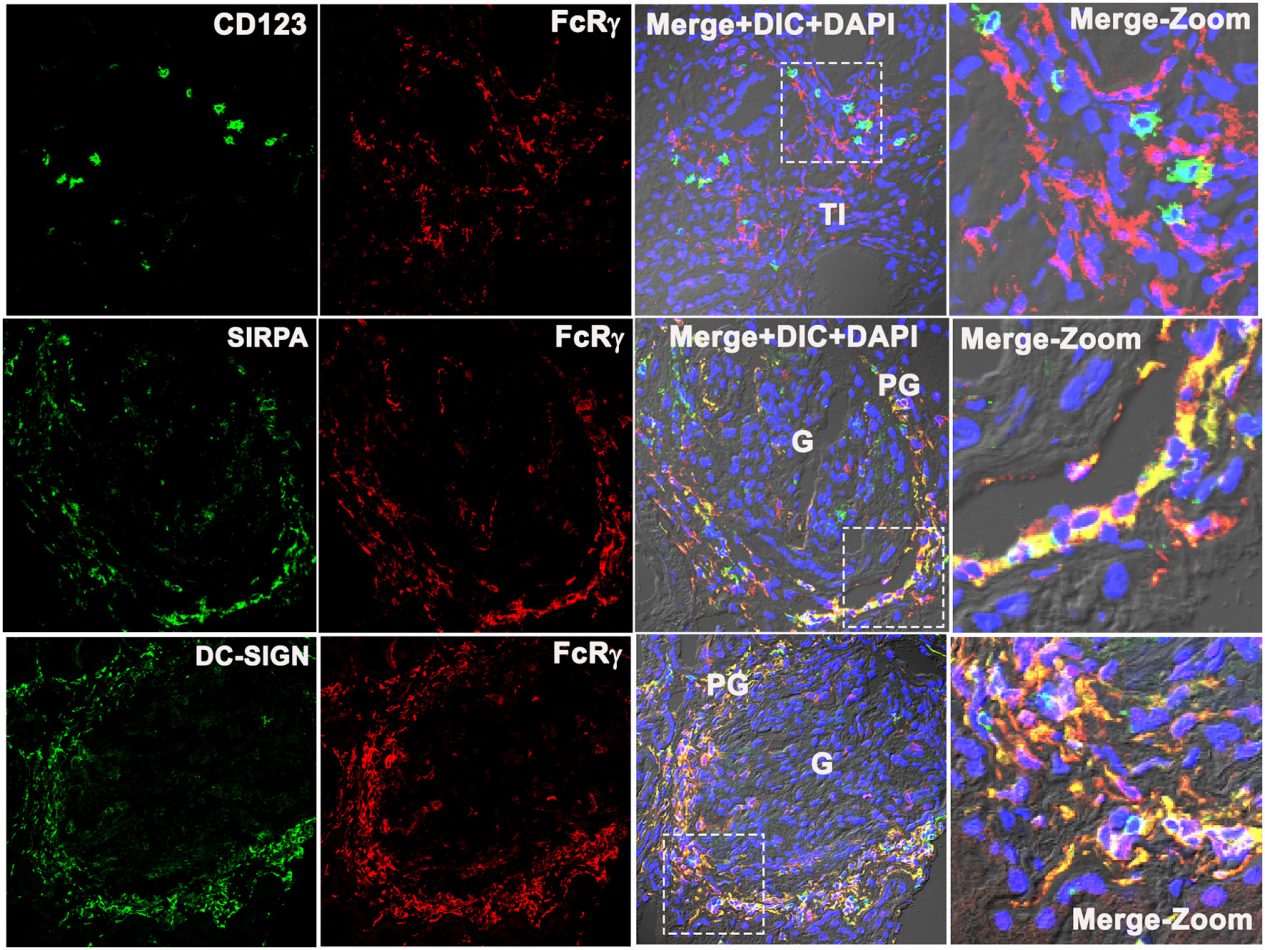
Fc receptor gamma chain does not colocalize with plasmacytoid DC marker CD123, but colocalizes with monocyte-derived DC marker SIRPA and DC-SIGN in the human LN kidney. Three color confocal IF images that are representative of 3 human LN kidneys showing no colocalization of CD123 (green) with Fc receptor gamma chain (FcRγ) (red) (top row) in the TI region of the kidney, but colocalize with monocyte-derived DC cell marker SIRPα (green) (middle row) and DC-SIGN (green) with FcRγ (red) (bottom row) in the PG region. The third column shows merged images of the first 2 columns plus DIC microscopy and DAPI staining of nuclei (blue). The fourth column shows the zoomed image of the third column highlighted using a dotted square.

To identify which subset of FcRγ-expressing DC is found in the PG region during LN flare, the biopsy tissue was stained for CD123, a specific marker of a plasmacytoid DC (pDC) (16, 17). CD123^+^ cells were found in the TI region (Figure 5, top row) but were absent from the glomeruli and the PG region of LN kidneys. CD123^+^ cells were sparse, and CD123 staining in the TI did not align with FcRγ staining. This suggests that the intrarenal FcRγ-expressing DC in LN is not a pDC.

Signal regulatory protein alpha (SIRPα) expression colocalized with FcRγ (Figure 5, middle row), suggesting that the PG DCs are not myeloid conventional type 1 DC (cDC1) (16). The remaining possibilities were that the PG DCs are myeloid conventional type 2 DCs (cDCs2) or monocyte-derived DCs (moDCs). To distinguish between these DC subsets, the DC-SIGN expression, a marker specific to moDCs was assessed. DC-SIGN colocalized with FcRγ in the PG region (Figure 5, bottom panel) suggesting that the PG DC is a moDC.

### FcRγ Colocalized With CD64, CD16, and CD14 in Periglomerular Region of Kidney and Confirmed That DCs Found in the LN Kidney at Flare Are moDCs

To confirm FcRγ expressing DCs are moDCs, they were further characterized by evaluating the presence of additional cell surface markers known to be present on the moDC including CD64, CD16, and CD14. Each of these markers was found in the LN kidney tissue and colocalized with FcRγ (Supplementary Figure 1).

### FcRγ Colocalized With Previously Identified Circulating infDC Marker CD163 and CD163 Expression Is Overexpressed at LN Flare

**Figure 6:**
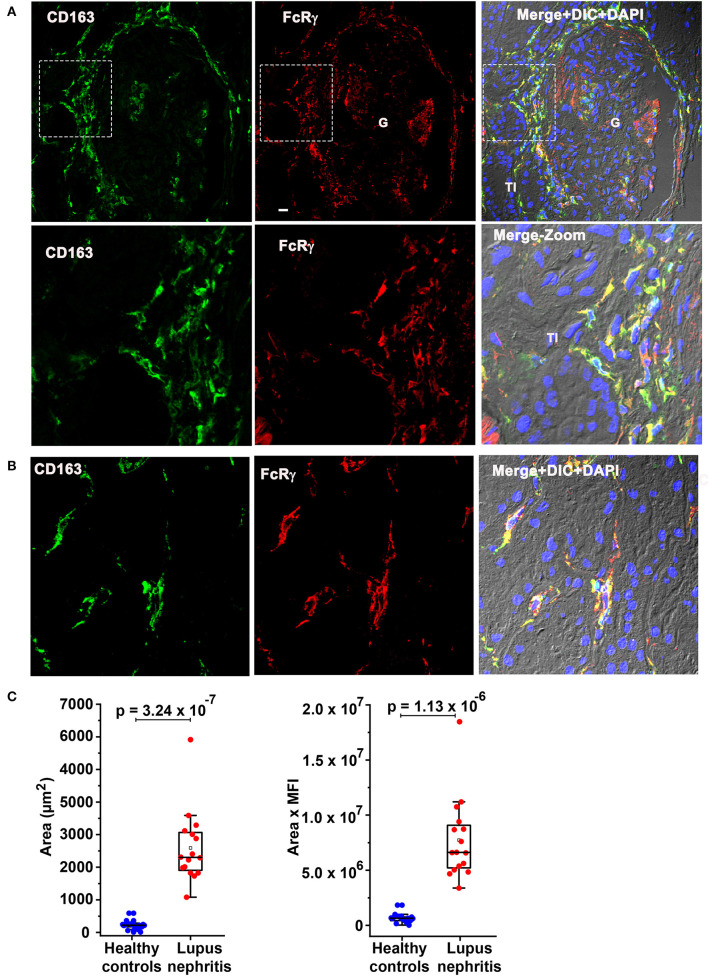
Fc receptor gamma chain colocalizes with previously identified circulating inflammatory dendritic cell (infDC) marker CD163 and CD163 expression is overexpressed in human LN compared to HCs. **(A)** Three color IF images that are representative of 3 human LN kidneys showing colocalization of CD163 in green (first) with FcRγ in red (middle) in the PG region. The second row shows a zoomed portion of images of the top row highlighted in the dotted square. The third column shows the merging of the first 2 along with DIC and DAPI staining of nuclei (blue). **(B)** Three color IF images that are representative of the 3 human LN kidneys showing colocalization of CD163 in green (first) with FcRγ in red (middle) in the tubulointerstitial (TI) region. **(C)** The bar graph portrays the quantification of infDCs based on CD163 expression using green color from anti-CD163 staining from 18 G images of 3 different HCs and 16 glomeruli images from 4 different LN samples using ImageJ software. The area alone (graph in left) and area × mean fluorescence intensity (graph in left) of CD163 (green) were measured and plotted with mean ± SD. The data were analyzed by unpaired *t*-test (one-tailed) using each measurement, and *p*-values were indicated in the bar graph.

Subsequently, colocalization analysis of CD163 with FcRγ was done to determine if the moDC population seen here could be the recently described circulating infDC (18). Figure 6 confirms CD163 colocalization (green) with FcRγ (red) in the PG region (Figure 6A) and the TI (Figure 6B) strongly suggesting that the moDC subset present in the kidney during LN flare are indeed infDCs. As infDCs do not express CD5 (18), the kidney tissue was stained for CD5. Although CD5-expressing cells were present in the kidney, they were not located in proximity to the PG region and did not colocalize with FcRγ (Supplementary Figure 2), suggesting that these infDC lack CD5 expression.

To quantitate the difference in infDC in LN and HC kidneys, infDC were quantified by measuring the area of CD163 expression after staining with an anti-CD163 monoclonal antibody, and the mean fluorescence intensity of CD163. Using 16 glomerular images from 4 LN flare kidneys, and 18 glomerular images from three HC kidneys, we found a 9- to 21-fold higher level of infDC in LN kidneys compared to HC (Figure 6C).

Since infDCs have previously been shown to express CD11b (19, 20), we further analyzed the human LN kidney with rat anti-human CD11b mAb (clone M1/70) along with anti-CD163 antibody. Consistent with previous findings in this manuscript (Figure 3), the clone M1/70 gave a weak-to-nil signal in the infDCs (Supplementary Figure 3).

### Inflammatory Dendritic Cells Were Present in Close Proximity With T Cells at LN Flare

**Figure 7:**
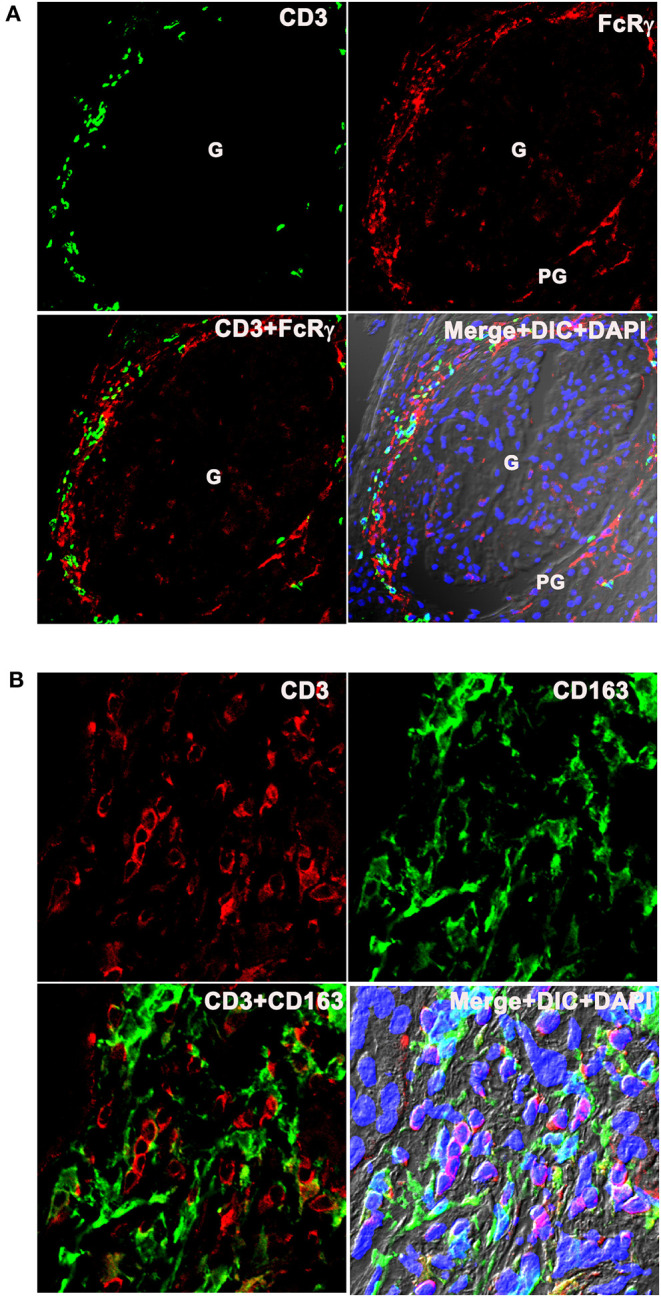
Inflammatory dendritic cells are present in close proximity with T cells at LN flare. **(A)** The top row portrays 3 color IF images that represents three LN kidneys showing the presence of CD3 (Pan T cell marker in green) next to FcRγ (infDC marker in red). The bottom row portrays the merged images of the first 2 columns in the right and merged images of the first 2 columns plus DIC and DAPI staining of nuclei (blue) in the left. **(B)** The top row portrays 3 color IF images that represent the three different LN kidneys showing the presence of CD3 (pan T cell marker in red) adjacent to CD163 (infDC marker in green) that are highly magnified from the PG region. The bottom row shows the merged images of the first 2 columns in the right and merged images of the first 2 columns plus DIC and DAPI staining of nuclei (blue) in the left.

Lupus nephritis kidney sections were costained with the T cell marker CD3, and antibodies to FcRγ and CD163 to spatially localize T cells and infDCs. CD3 costaining with infDC markers were done in two ways using two different clones of anti-CD3 antibodies (mouse mAb UCHT1 and rabbit mAb SP7) and 2 infDC markers. The CD3 mAb UCHT1 and anti-FcRγ staining showed the presence of CD3^+^ T cells present adjacent to FcRγ^+^ infDCs in the PG region of the LN kidney (Figure 7A). Staining with anti-CD163^+^ CD3 mAb SP7 (Figure 7B) confirmed that infDC were present in close proximity to CD3^+^ T cells in the human LN kidney. Although the costaining of CD3 with infDC markers showed mainly discrete green and red staining for both cell types, in a few places the red and green staining overlapped.

## Discussion

In this study, we identified a novel infDC population infiltrating the kidney at the LN flare. These infDCs infiltrate, localize to the PG and TI spaces, and their expression was 10-fold greater in the LN flare kidney compared to the HCs. The evidence that these are novel is based on their surface markers, being FcRγ^+^, MHCII^+^, CD11c^+^, CD163^+^, CD5^−^, DC-SIGN^+^, CD64^+^, CD14^+^, CD16^+^, SIRPα^+^, CD206^−^, CD68^−^, CD123^−^, CD3^−^, and CD11b^−^. Notably, these infDC were present in close approximation to T cells in the PG region of the kidney during LN flare.

The observations from this investigation are supported by previous studies that detected PG DC infiltration in various mouse models of experimental glomerulonephritis (21, 22). However, it is unclear why the infDC settle in the PG space. The previous literature suggests that the renal DC starts in the glomerulus and traverse from mesangium through glomerular tuft and lymphatics to draining lymph nodes in order to present antigen to T cells in the PG (23). In this case, the renal DC may start in the glomerulus but by the time LN becomes clinically evident, these DC have already moved out of the glomerulus and settled in the PG space. Inhibitory signals may then be released and block the cytokine/chemokines required for monocyte differentiation into the infDC, explaining the absence of infDCs from the LN glomeruli at the time of biopsy. Alternatively, it is possible that DC chemotactic factors are released directly from renal tubular cells attracting infiltrating DC (24). We speculate that both occur in response to inflammatory stimuli resulting in PG and TI accumulation of infDC during LN.

This investigation established that the PG DC are not plasmacytoid or conventional DCs. The expression of monocyte markers CD14, CD64, and CD16 that were reported earlier, confirmed that these PG DCs represent a moDC (16, 25, 26). In the setting of active inflammation or infection, moDCs are termed infDCs (27). Further characterization of these moDCs revealed a unique infDC population not previously described in the human LN. Notably, however, a recent transcriptomic study of peripheral blood immune cells in patients with SLE identified an infDC signature that was CD163^**+**^, CD14^**+**^, and CD5^**−**^, which is similar to the intrarenal infDC population reported here (18). Recently, an extensive evaluation of immune cells was performed using single-cell RNA-Seq from kidney biopsies at the LN flare (28). Several monocyte clusters were identified in this study with one monocyte cluster expressing a CD163^**+**^, CD14^**+**^, CD16^**+**^, CD64^**+**^, and DC-SIGN^**+**^ signature, similar to the infDC described here. Furthermore, the characterized monocyte lineage was thought to be an infiltrating monocyte subset as it was minimally expressed in healthy kidneys. While these IF studies do not provide a precise measure of the level of lineage marker expression, and this could make identification of transitional stages of the monocyte to DC conversion difficult, these findings do allow for characterization and spatial orientation to inform further quantitative studies.

This novel infDC population also resembles previously reported infDC, from lymph nodes of Listeria-infected mice (CD64^**+**^CD11c^+^MHCII^**+**^) (27), and gut mucosa of celiac disease (29). InfDCs have also previously been described in human arthritic synovial fluid from patients with rheumatoid arthritis (20). However, the infDC described in the LN kidney differs from synovial fluid infDC; in that, they lack the macrophage markers CD11b and CD206 that are present on synovial fluid infDC. This suggests that the infDC population described here differs from the infDC seen in at least some other autoimmune diseases and may be unique to the kidney during LN flare. Moreover, earlier studies have identified FcεRI [(19, 30); identified using an anti-FcεRIα antibody (eBioscience, Inc., San Diego, CA, USA) for antibody binding site] to be the best marker to distinguish infDC from macrophages and cDC by flow cytometry. Our studies suggest that FcRγ, a cytoplasmic gamma subunit through which multiple FcRs function, including FcεRI, FcγRI, FcγRIIIa, and FcαRI, as well as other immune receptors like GPVI, OSCAR, and TREM (8), to be a reliable marker for IF studies on patient biopsy samples. It is likely that infDC in the LN kidney express FcεRI, similar to FcγRI and FcγRIIIa (Supplementary Figure 1) since the presence of FcRγ indirectly suggests the presence of multiple receptors including FcεRI.

The importance of characterizing the intrarenal cell type expressing CD163 is enhanced by recent findings from our group, revealing that urine CD163 levels were significantly higher in active LN compared to extrarenal SLE or inactive SLE (31). Urine CD163 correlated with disease severity and histologic activity index. While the study by Mejia et al (31). suggested CD163 derives from M2 macrophages, we now suggest that urine CD163 also derives from infDC, supporting the idea that urine CD163 is a biomarker that reflects disease activity in LN.

With regard to the origin of these infDCs, the expression of monocyte markers suggests that infDCs may be differentiated from infiltrated monocytes in the kidney during inflammation caused by various autoimmune stimuli including immune complexes. On the other hand, the presence of CD163^+^CD14^+^ infDCs in the patients with circulation of lupus (18) suggests that infDC from the peripheral circulation enter the kidney in LN. It is also possible that both mechanisms account for renal infDCs.

The mechanisms by which infDC interact with T cells during human LN are currently unknown. Although the costaining of CD3 along with infDC markers shows mainly discrete populations of both cell types in close proximity (Figure 7B), there is also evidence of some overlap suggesting immunological synapse formation and interactions between these infDCs and T cells in the LN kidney. However, T cells are poised to migrate to secondary lymphoid organs; a recent report has shown the presence of immune aggregates organized as tertiary lymphoid structures (TLS) in patients with LN and murine LN kidneys that resemble lymph nodes by gene signatures and cell composition (32). This is consistent with a previous report that identified germinal centers with T- and B-cell aggregates in the LN kidney (33). Considering these findings in the context of identifying infDCs in the LN kidney suggests that infDCs in the PG and TI may be important drivers of a local adaptive immune response within the LN kidney.

While it is not yet clear whether these T cells are resident T cells or primed T cells, infDCs are known under various disease conditions to have the capacity to trigger the development of major T helper cell subsets, namely Th1, Th2, and Th17 (20, 34, 35). Although, *in vitro* studies suggest infDCs are involved in the initiation and maintenance of Th17 response (19), further studies on the novel LN kidney infDCs are needed because the inflammatory stimulus and the tissue microenvironment determine infDC function *in situ* (36).

## Conclusion

In conclusion, we identified a novel infDC population that has not been previously described in the LN kidney. These infDCs reside in the PG region and adjacent to infiltrating T cells. Our findings, coupled with recent literature identifying circulating CD163^+^ infDC in SLE and urine CD163 as a valuable marker of disease activity in LN, suggest infDCs and their T cell partners may be key contributors to driving the local adaptive immune response during active LN. Further study is necessary to define the T cell subsets residing next to the infDCs and understand the mechanisms by which PG infDCs communicate with T cells to drive local inflammation in LN.

## Data Availability Statement

The raw data supporting the conclusions of this article will be made available by the authors, without undue reservation.

## Ethics Statement

The studies involving human participants were reviewed and approved by IRB. The patients/participants provided their written informed consent to participate in this study.

## Author Contributions

SP, JS, JT, HS, and LG conducted the experiments. AM, VA, BL, JM-V, and AS contributed to the patient's samples. SP, DB, WJ, BR, and LG contributed to the design of the study and analyzed the data. JZ and LY performed statistical analysis on the nanostring data. SM contributed reagents and performed the statistical analysis. LG, SP, and BR wrote the manuscript. SP and LG conceived the study. All authors contributed to manuscript revision, read, and approved the submitted version.

## Conflict of Interest

The authors declare that the research was conducted in the absence of any commercial or financial relationships that could be construed as a potential conflict of interest.
