# Supplementary material for: A Novel Inflammatory Dendritic Cell That Is Abundant and Contiguous to T Cells in the Kidneys of Patients With Lupus Nephritis
Source: Front Immunol. 2021 Feb 17;12:621039. doi: 10.3389/fimmu.2021.621039 (PMC7919935; doi:10.3389/fimmu.2021.621039)
Supplement: Supplementary file 1 [file Data_Sheet_1.PDF]

**Antigen[a]**	**Host[b]**	**Clone[c]**	**Source[e]**
**FcεRI γ subunit**	**Rabbit pAb**	**polyclonal**	**EMD Millipore and Invitrogen**
**CD11c**	**rabbit mAb**	**monoclonal**	**Abcam**
**CD3**	**mouse mAb**	**UCHT1**	**Biolegend**
**CD206**	**goat pAb**	**polyclonal**	**Santa Cruz**
**CD206**	**rabbit pAb**	**polyclonal**	**Santa Cruz**
**CD11b**	**mouse mAb**	**ICRF 44**	**Biolegend**
**CD11b**	**rat mAb**	**M1/70**	**Biolegend**
**CD11c**	**mouse mAb**	**3.9**	**Biolegend**
**CD3**	**rabbit mAb**	**SP7**	**Novus Biologicals**
**CD209/DC-SIGN**	**mouse mAb**	**9E9A8**	**Biolegend**
**CD123**	**mouse mAb**	**6H6**	**eBioscience**
**CD74/MHCII**	**mouse mAb**	**LN2**	**Biolegend**
**CD16**	**mouse mAb**	**5B11**	**Novus Biologicals**
**CD64**	**mouse mAb**	**10.1**	**Emd Millipore**
**CD14**	**mouse mAb**	**M5E2**	**Biolegend**
**CD163**	**mouse mAb**	**GHI/61**	**Biolegend**
**CD163**	**mouse mAb**	**RM3/1**	**Biolegend**
**CD5**	**mouse mAb**	**UCHT2**	**Biolegend**
**CD172a (SIRPα)**	**mouse mAb**	**P84**	**Biolegend**

Sup Table 1: Details of all primary antibodies used in this study

[a] Molecule or epitope recognized by antibody. [b] The host species in which the antibodies were made. Abbreviations: mAb, monoclonal antibody; pAb, polyclonal antibody[c]. The clone name of the antibody[c].

DE Glom. Transcripts at LN Flare vs Normal			DE TI Transcripts at LN Flare vs Normal		
Name	Fold Change	P	Name	Fold Change	P
FCER1G	3.5	0.0001	*C1R*	1.9	1E-10
SPP1	3.1	2.00E-04	*STAT1*	1.8	0.00000001
CD45R0	2.9	0	*MX1*	1.8	4.00E-04
C1QB	2.4	0	*CCL19*	1.8	0.001
CCL2	2.2	0.001	*PTPRC_all*	1.8	1.00E-04
FN1	2.0	0.006	***FCER1G***	1.7	0.001
MSR1	1.9	0	*C2*	1.7	0.00000005
TGFBI	1.9	0.004	*LTF*	1.7	7.00E-04
CD45RB	1.9	1.00E-04	*ITGA4*	1.7	0.0000001
CTSS	1.9	0	*CCL5*	1.6	9.00E-04
CYBB	1.8	0	*IFI35*	1.6	0.003
MX1	1.8	0.002	*IFI16*	1.6	0.000001
ITGAX	1.7	0	*C3*	1.6	8.00E-04
C1QA	1.7	1.00E-04	*C1QB*	1.6	1.00E-04
PTPRC_all	1.7	6.00E-04	*C1S*	1.5	6.00E-04
ITGAM	1.7	1.00E-04	*C1QA*	1.5	0.0000005
CFD	1.6	1.00E-04	***ITGAX***	1.5	2.00E-04
S100A9	1.6	1.00E-04	*CD45R0*	1.5	0.02
POU2F2	1.6	2.00E-04	*TAP1*	1.5	0.004
TLR2	1.6	1.00E-04	*TGFBI*	1.5	0.02
C2	1.6	2.00E-04	*IFNAR2*	1.5	0.000002
ITGB2	1.6	4.00E-04	*CFD*	1.5	3.00E-04
IL10RA	1.6	0	*HLA-A*	1.5	7.00E-04
SYK	1.6	0.0106	*IRGM*	0.6	0.01
TNFSF8	1.6	4.00E-04	*DEFB1*	0.6	0.00005
CD44	1.5	0.0013	*THY1*	0.6	2.00E-04
IKBKE	1.5	0.0213	*EGR1*	0.6	0.009
ITGAL	1.5	6.00E-04	*RORC*	0.6	0.00007
IFNA2	0.7	0.0372	*KIT*	0.6	9.00E-04
ITGA2B	0.7	0.0065	*MME*	0.5	0.00001
RORC	0.6	6.00E-04	*ZBTB16*	0.5	0.03
MME	0.5	2.00E-04			
CR1	0.5	0			

Sup Table 2. Differentially Expressed Glomerular and Tubulointerstitial transcripts at LN Flare vs Healthy Controls

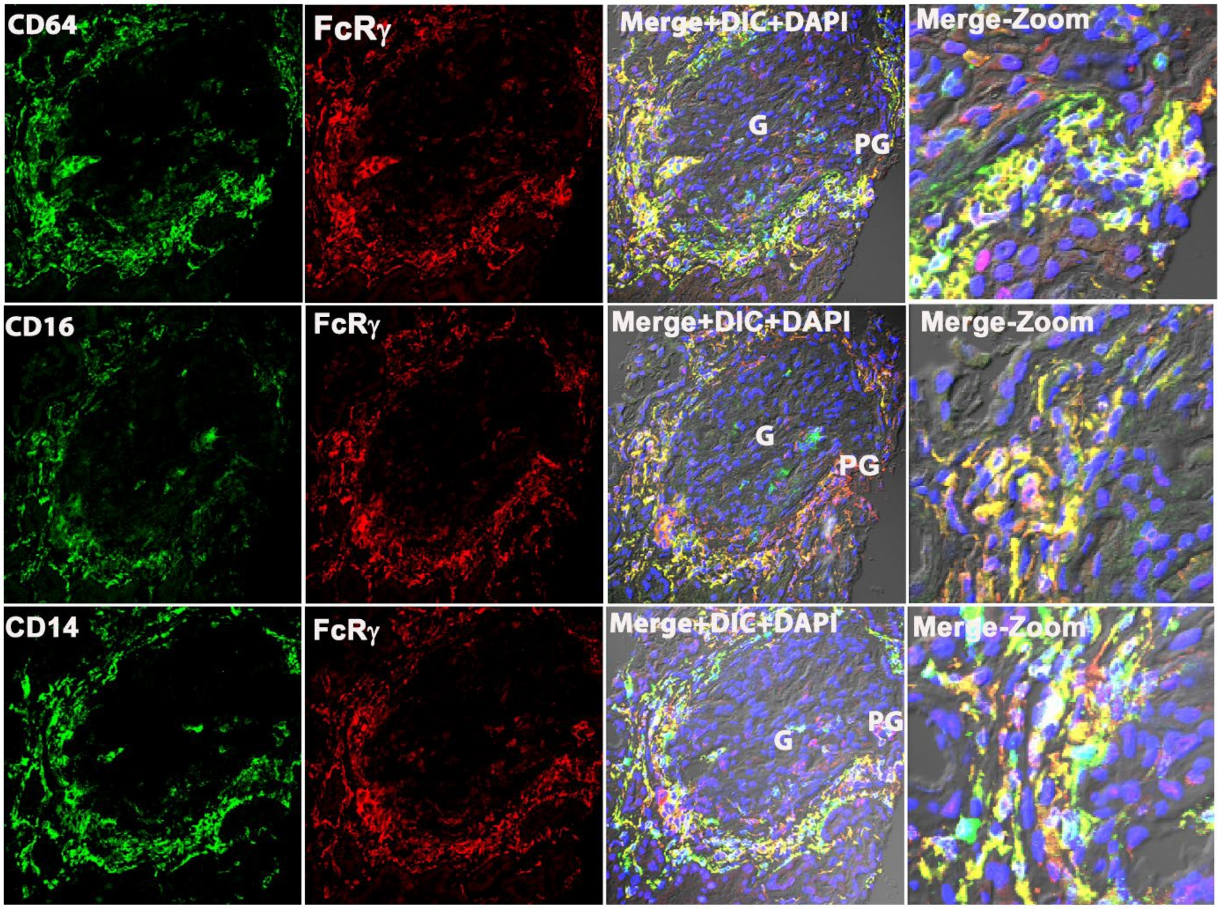

Sup Figure 1: FcRγ colocalize with CD64, CD16 and CD14 in periglomerular region of kidney showing the presence of inflammatory dendritic cells. Three color confocal Immunofluorescence images of human Lupus Nephritis kidney showing colocalization of CD64(green) with FcRγ (Red) (Top row), CD16 (green) with FcRγ (red) (middle row) and with CD14 (green) with FcRγ (red) in the bottom row in the periglomerular region. The Column 3 shows merged images of the first two columns plus Differential interference contrast (DIC) microscopy and DAPI staining of nuclei (blue). PG, periglomerular region; G, Glomeruli.

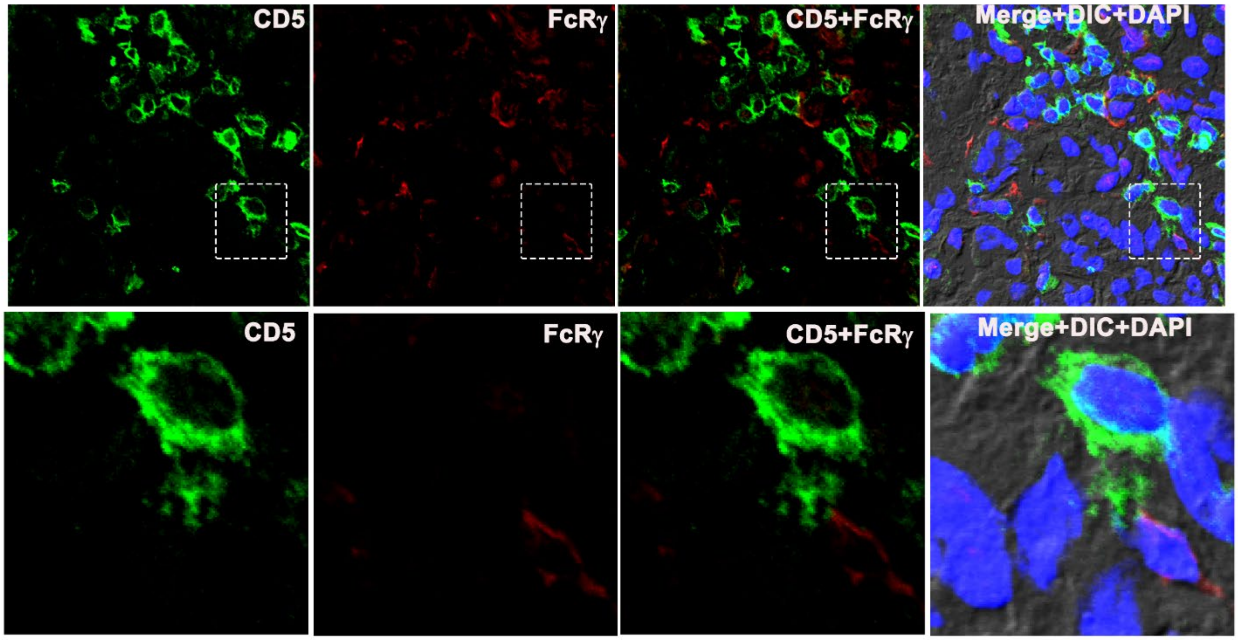

Sup Figure 2: FcRγ expressing infDC doesn't express CD5. **a.** The top row portrays 3 color IF images that represents 2 Lupus nephritis kidney showing the expression of CD5 (green) and FcRγ (infDC marker in red). The third column shows the merged image of green and red. The fourth column shows the merged image in column 3 plus DIC and DAPI (blue) staining of the nuclei. The bottom row higher power images of the top row.

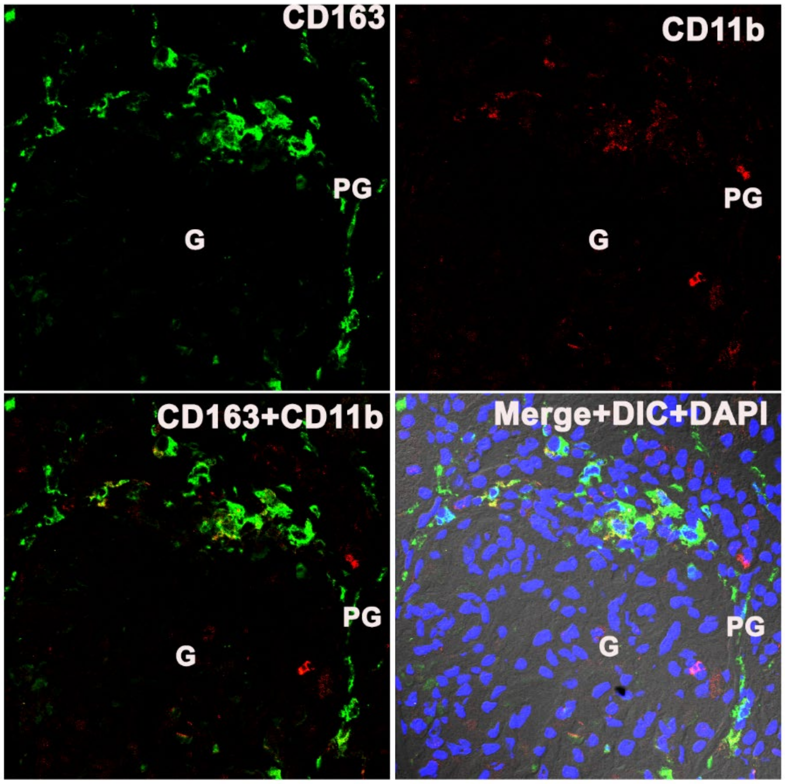

Sup Figure 3: CD11b is weakly expressed in infDC of human LN kidney a. The top row portrays 3 color IF images that represents 3 LN human kidneys showing presence of CD163 (infDC marker in green) with CD11b (red). The bottom row portrays the merged images of the top row in the right and merged images of the top row plus Differential interference contrast (DIC) microscopy and DAPI staining of nuclei (blue) in the left. PG, periglomerular region; G, Glomeruli.

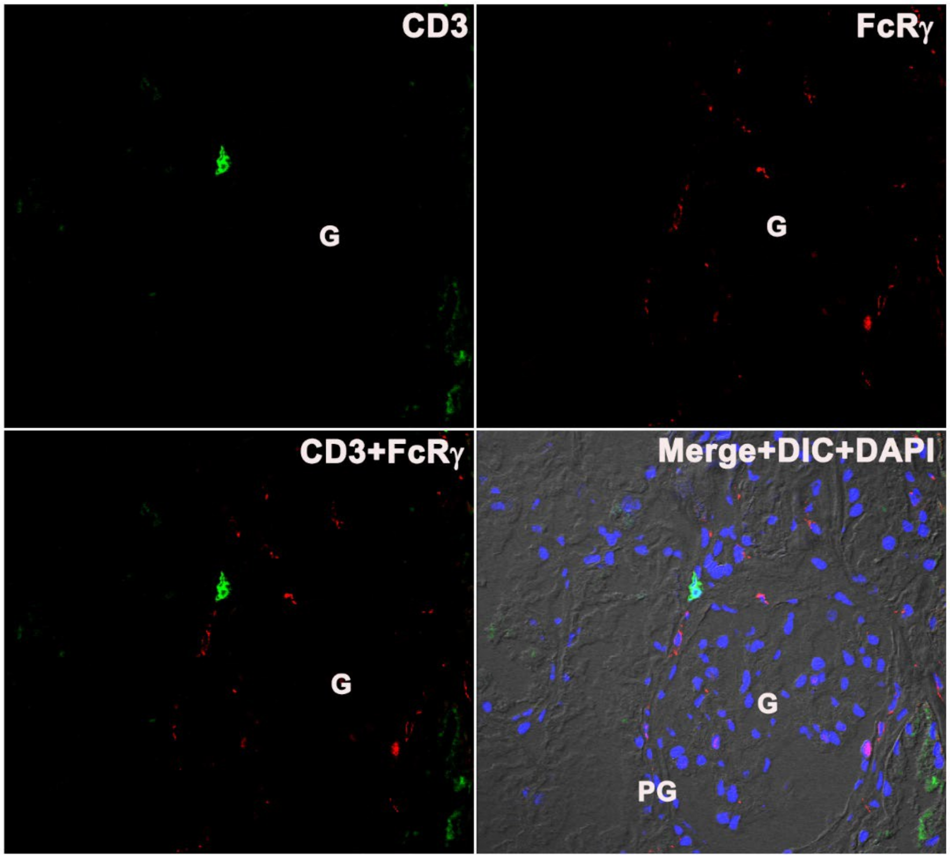

Sup Figure 4: FcRγ expressing infDC and CD3 expressing T cells are sparsly present in the PG region of healthy human kidney. **a.** The top row portrays 3 color IF images that represents 3 healthy human kidneys showing presence of CD3 (Pan T cell marker in green) next to FcRγ (infDC marker in red). The bottom row portrays the merged images of the top row in the right and merged images of the top row plus Differential interference contrast (DIC) microscopy and DAPI staining of nuclei (blue) in the left. PG, periglomerular region; G, Glomeruli.
